# Clinical features and long-term outcomes of interstitial lung disease with anti-neutrophil cytoplasmic antibody

**DOI:** 10.1186/s12890-021-01451-4

**Published:** 2021-03-16

**Authors:** Xin Sun, Min Peng, Ting Zhang, Zongru Li, Lan Song, Mengtao Li, Juhong Shi

**Affiliations:** 1 Department of Respiratory and Critical Care Medicine, Peking Union Medical College Hospital, Chinese Academy of Medical Science and Peking Union Medical College, No. 1 Shuai Fu Yuan, Dongcheng District, Beijing, 100730 China; 2 Peking University Institute of Haematology, Peking University People’s Hospital, No. 11 Xizhimen South Street, Beijing, 100044 China; 3 Department of Radiology, Peking Union Medical College Hospital, Chinese Academy of Medical Science and Peking Union Medical College, No. 1 Shuai Fu Yuan, Dongcheng District, Beijing, 100730 China; 4 Department of Rheumotology, Peking Union Medical College Hospital, Chinese Academy of Medical Science and Peking Union Medical College, No. 1 Shuai Fu Yuan, Dongcheng District, Beijing, 100730 China

**Keywords:** Anti-neutrophil cytoplasmic antibody, Interstitial lung disease, Microscopic polyangiitis, Idiopathic interstitial pneumonia

## Abstract

**Background:**

Patients with interstitial lung disease (ILD) are occasionally positive for anti-neutrophil cytoplasmic antibodies (ANCAs). Differences between ILDs secondary to microscopic polyangiitis (MPA) and isolated ANCA-positive idiopathic interstitial pneumonia (IIP) remain unclear. The aim of this study was to explore the differences in clinical features and outcomes between MPA-associated ILDs and isolated ANCA-positive IIPs.

**Methods:**

We reviewed 1338 ILDs patients with available ANCA results and retrospectively analysed 80 patients who were ANCA-positive. MPA-associated ILDs (MPA-ILDs group) and isolated ANCA-positive IIPs (ANCA-IIPs group) were compared.

**Results:**

Among 80 patients with ANCA-positive ILDs, 31 (38.75%) had MPA-ILDs, and 49 (61.25%) had isolated ANCA-positive IIPs. Compared with ANCA-IIPs group, patients in MPA-ILDs group had a higher proportion of fever (*p* = 0.006) and higher neutrophil count (*p* = 0.011), erythrocyte sedimentation rate (ESR) (*p* < 0.001) and C-reactive protein (CRP) (*p* = 0.005). Multivariable analysis showed that ESR level was an independent risk factor for mortality in all 80 ANCA-positive ILDs patients (HR 1.028, *p* = 0.001). Survival in MPA-ILDs group was lower than that in ANCA-IIPs group, and further stratified analysis revealed that ANCA-IIPs patients with elevated ESR or CRP had a worse prognosis than those with normal inflammation markers, with 5-year cumulative survival rates of 60.00%, 86.90% and 100.00% in MPA-ILDs and ANCA-IIPs with and without elevated inflammation markers, respectively.

**Conclusions:**

Among patients with ANCA-positive ILDs, the prognoses of ANCA-IIPs with normal inflammation markers, ANCA-IIPs with elevated inflammation markers and MPA-ILDs were sequentially poorer. Therefore, stratified treatment should be considered in the management of ILDs patients positive for ANCAs.

**Supplementary Information:**

The online version contains supplementary material available at 10.1186/s12890-021-01451-4.

## Background

Interstitial lung disease (ILD) is a heterogeneous group of parenchymal lung disorders of variable aetiologies. The diagnosis of idiopathic interstitial pneumonia (IIP) must exclude known causes, especially connective tissue disease (CTD). Therefore, CTD-related clinical manifestations and autoantibodies should be fully evaluated in ILDs patients, and those who meet the CTD diagnostic criteria are referred to as CTD-ILDs [1]. Patients who have positive serologies but do not fulfil the classification criteria of a given CTD have been classified as a newly proposed disease entity called interstitial pneumonia with autoimmune features (IPAF) [2]. The possible connection between anti-neutrophil cytoplasmic antibody (ANCA) and ILDs has been reported in a number of studies in the past few years [3, 4]. However, ANCA is not included in the current recommendations for serologic evaluation in patients with IIPs, CTD-ILDs, or IPAF [1, 2, 5].

ANCAs are a family of autoantibodies that react with antigens located in the cytoplasmic granules of neutrophils and the lysosomes of monocytes. Myeloperoxidase (MPO) and proteinase 3 (PR3) are two major ANCA antigens, and autoantibodies with specificity for the two antigens are referred to as MPO-ANCA and PR3-ANCA, respectively [6]. ANCA-associated vasculitis (AAV) is a collective term for multi-systemic, necrotizing vasculitis that primarily affects small vessels, including microscopic polyangiitis (MPA), granulomatosis with polyangiitis (GPA), and eosinophilic granulomatosis with polyangiitis (EGPA) [7]. ANCAs have been recognized as crucial markers for the diagnosis and monitoring of these diseases. ILDs has been regarded as a major form of pulmonary manifestation of AAV, especially of MPA [8–10].

While AAV-associated ILDs (AAV-ILDs) refers to ILDs patients with serum ANCA positivity and extrapulmonary vasculitic involvement that meet the diagnostic criteria of AAV, some ILDs patients present with ANCA positivity without extrapulmonary manifestations or histopathological evidence for vasculitis. This group of patients can be classified as having either idiopathic pulmonary fibrosis (IPF) or IIP since ANCA is not included in the current diagnostic algorithm for ILDs [5, 11, 12]. It is unclear whether ILDs with isolated ANCA positivity behaves more like AAV-ILDs or IIP/IPF. Another problem concerns whether those patients should be treated as if they had AAV-ILDs or IIP/IPF. Previous studies have compared ANCA-positive and ANCA-negative IPF patients, with inconsistent findings regarding the prognostic impact of ANCAs on IPF [13–15]. However, limited studies have directly compared ILDs with isolated ANCA positivity with AAV-ILDs, and differences between these two groups remain unclear.

Therefore, our study retrospectively analysed ILDs patients positive for ANCAs. By comparing the diagnosed AAV-ILDs patients with isolated ANCA-positive IIPs patients, we aimed to identify the differences in their clinical and prognostic characteristics to further guide clinical management.

## Methods

### Study population

**Fig. 1:**
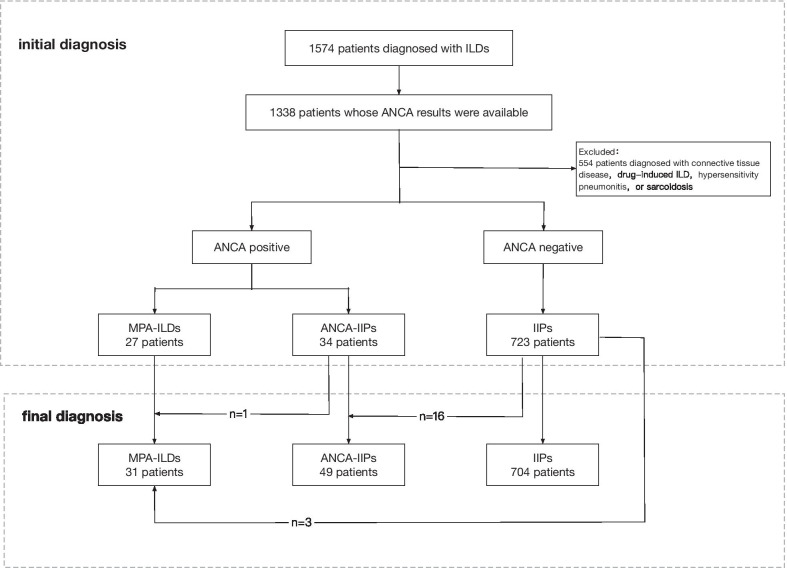
Flowchart of patient screening and classification. ILD: interstitial lung disease; ANCA: anti-neutrophil cytoplasmic antibody; MPA: microscopic polyangiitis; IIP: idiopathic interstitial pneumonia

We retrospectively reviewed patients who had been diagnosed with ILDs at Peking Union Medical College Hospital (PUMCH) between June 2006 and July 2018. To be included in this study, patients were required to 1. Have a diagnosis of ILD based on clinical symptoms and radiologic features, with or without histopathologic results. Radiological features in chest high-resolution computed tomography (HRCT) include diffuse ground-glass opacities, reticular opacities or consolidation, with or without honeycombing and traction bronchiectasis [16]. 2. have available ANCA testing results during the first visit and follow-up period. Patients with ILDs secondary to CTD, drug, environment, or occupational exposure, hypersensitivity pneumonitis, and sarcoidosis were excluded from the study. Figure 1 presents the flowchart of patient screening and classification for this study. Patients with negative ANCA testing were defined as the IIPs group. In all the patients with ANCA-positive ILDs, those who were diagnosed with MPA according to 2012 Chapel Hill consensus criteria [7] were defined as the MPA-ILDs group, and patients with isolated ANCA positivity who did not fulfil the MPA criteria were classified as the ANCA-IIPs group. Demographic characteristics, clinical manifestations, radiologic features and prognosis were compared between these two groups. All the diagnoses and classifications were applied after multidisciplinary team (MDT) discussions of all available information. This study was approved by the Peking Union Medical College Hospital Institutional Review Board (Reference Number: ZS-1054).

### Data collection

Baseline information at the time of initial diagnosis was obtained, and the following items were analysed: demographic information (age, gender), clinical course, clinical symptoms and signs, laboratory findings (routine blood and urine, liver and renal function tests, erythrocyte sedimentation rate [ESR], C-reactive protein [CRP], rheumatoid factor and serologic autoantibodies), pulmonary function tests (PFTs), and chest HRCT scans.

ANCA was assessed by indirect immunofluorescence, while MPO-ANCA and PR3-ANCA titres were measured by ELISA.

Chest HRCT images were evaluated by at least two pulmonologists and radiologists. The HRCT scans were analysed for the following characteristics: ground-glass opacities, consolidation, reticular patterns, honeycombing, traction bronchiectasis, interlobular septal thickening, pleural thickening, etc.

Treatment regiments were recorded. The patients were followed up at least once a year, and basic laboratory tests, serologic autoantibodies, PFTs and chest HRCT were evaluated routinely. The follow-up period ended in April 2019, and the outcomes were defined as death from all causes and lung transplantation.

### Statistical analysis

All data were analysed using statistical analysis software (SPSS version 24.0, IBM Corporation). Normally distributed variables are presented as the means ± SDs, and Student’s t test was used for comparisons. Continuous, non-normally distributed data are presented as medians with interquartile ranges (IQRs), and the Mann–Whitney U test was used for comparisons. Categorical variables are expressed as numbers (%), and the *χ*2 test or Fisher’s exact test was used for comparisons. Survival analysis was performed by Kaplan–Meier analysis using the log-rank test. Cox models were used to examine the association between baseline characteristics and mortality. *P* values are two-sided, and *P* < 0.05 was considered statistically significant.

## Results

A total of 1574 patients were diagnosed with ILDs, among whom 1338 had available ANCA testing results. Sixty-one patients had positive ANCA findings at first diagnosis, composed of 27 patients in MPA-ILDs group and 34 in ANCA-IIPs group. 61/1338 (4.60%) patients with ILDs and 34/757 (4.50%) patients with IIPs were ANCA-positive. During follow-up, among the 723 IIPs patients with negative ANCA findings, 19 (2.63%) converted to ANCA positive, with a median conversion period of 16 (IQR, 7–24) months. A total of 2.94% of the ANCA-IIPs group and 0.41% of the IIPs group developed MPA during follow-up. By April 2019, a total of 80 patients were enrolled in the ANCA-positive cohort, including 31 (38.75%) in the MPA-ILDs group and 49 (61.25%) in the ANCA-IIPs group. A total of 80/1338 (5.98%) patients with ILDs and 49/757 (6.47%) patients with IIPs were ANCA-positive (Fig. 1).

### Demographic and clinical characteristics

Eighty patients (36 males [45.00%], 44 females [55.00%]) with ANCA-positive ILDs were included in the cohort. The median age at first diagnosis was 60 (IQR 51–66) years. There were no significant differences in age or gender between ANCA-IIPs group and MPA-ILDs group (Table 1).

**Table 1:** Demographic and clinical characteristics of patients with ANCA-IIPs versus patients with MPA-ILDs

	Combined (N = 80)	ANCA-IIPs (N = 49)	MPA-ILDs (N = 31)	*p* value
Age, y	60 (51, 66)	59 (52, 65)	62 (47, 67)	0.812
Male	36 (45.00%)	24 (48.98%)	12 (38.71%)	0.489
Follow-up time, m	40 (27, 58)	41 (28, 64)	36 (21, 52)	0.230
*Symptoms*				
Fever	26 (32.50%)	10 (20.41%)	16 (51.61%)	0.006*
Cough	70 (87.50%)	42 (85.71%)	28 (90.32%)	0.733
Dyspnoea	49 (61.25%)	29 (59.18%)	20 (64.52%)	0.648
Fatigue	11 (13.75%)	5 (10.20%)	6 (19.35%)	0.322
Arthralgia	23 (28.75%)	15 (30.61%)	8 (25.81%)	0.801
*Signs*				
Crackling	45 (56.25%)	26 (53.06%)	19 (61.29%)	0.497
Rash	10 (12.50%)	7 (14.29%)	3 (9.68%)	0.733
Clubbing fingers	7 (8.75%)	2 (4.08%)	5 (16.13%)	0.102
Mechanics hand	2 (2.50%)	2 (4.08%)	0 (0.00%)	0.519
Gottron’s sign	3 (3.75%)	2 (4.08%)	1 (3.23%)	1.000
*Systematic manifestations*				
Renal	30 (37.50%)	0 (0.00%)	30 (96.77%)	< 0.001*
Nervous system	3 (3.75%)	0 (0.00%)	3 (9.68%)	0.055
Cardiovascular	2 (2.50%)	0 (0.00%)	2 (6.45%)	0.147
Retinal	1 (1.25%)	0 (0.00%)	1 (3.23%)	0.388
*Treatment*				
Corticosteroid	76 (95.00%)	45 (91.84%)	31 (100.00%)	0.154
Cyclophosphamide	32 (40.00%)	15 (30.61%)	17 (54.84%)	0.038*
Others^#^	2 (2.50%)	2 (4.08%)	0 (0.00%)	0.519
None	2 (2.50%)	2 (4.08%)	0 (0.00%)	0.519
*Outcome*				
Death of all cause	15 (18.75%)	5 (10.20%)	10 (32.26%)	0.019*
Lung transplantation	1 (1.25%)	0 (0.00)	1 (3.22%)	0.388

Data are presented as the medians (IQRs), means ± SDs or No. (%)

^*^p < 0.05

^#^ inhaled corticosteroid

ANCA: anti-neutrophil cytoplasmic antibody; IIP: idiopathic interstitial pneumonia; MPA: microscopic polyangiitis; ILD: interstitial lung disease

The major symptoms and signs among our cohort patients included cough (87.50%), dyspnoea (61.25%), crackling (56.25%) and fever (32.50%). The frequency of fever tended to be higher in the MPA-ILDs group (51.61% vs 20.41%, *p* = 0.006). There were no differences in other symptoms and signs.

### Laboratory tests

Table 2 shows the laboratory test results of the 80 patients. Compared with those in the ANCA-IIPs group, neutrophil levels (6.79 vs 4.68, *p* = 0.011), ESR (69 vs 17, *p* < 0.001) and CRP (23.40 vs 2.44, *p* < 0.001) were significantly higher in the MPA-ILDs group.

**Table 2:** Laboratory tests and pulmonary function test findings of patients with ANCA-IIPs versus patients with MPA-ILDs

	Combined (N = 80)	ANCA-IIPs (N = 49)	MPA-ILDs (N = 31)	*p* value
*Laboratory findings*				
WBC, × 10^9/L	8.54 (6.18, 10.36)	7.44 (5.95, 9.75)	9.34 (7.15, 13.56)	0.061
NEUT, × 10^9/L	5.65 (3.97, 7.81)	4.68 (3.76, 6.94)	6.79 (5.06, 9.00)	0.011*
LY, × 10^9/L	1.90 (1.42, 2.36)	2.00 (1.55, 2.33)	1.47 (1.06, 2.41)	0.045*
HGB, g/L	132 (116, 147)	137 (130, 149)	116 (95, 139)	< 0.001*
PLT, × 10^9/L	246 (186, 290)	234 (176, 284)	251 (202, 322)	0.133
ESR, mm/h	53 (17, 87)	28 (13, 62)	86 (49, 99)	< 0.001*
CRP, mg/dL	15.92 (3.88, 53.70)	13.87 (2.21, 30.42)	35.77 (5.67, 71.18)	0.005*
PaO2, mmHg	75.9 (69.8, 82.2)	77.4 (72.3, 82.2)	71.5 (64.5, 83.0)	0.134
Cr (E), μmol/L	68 (57, 79)	67 (57, 74)	73 (61, 104)	0.035*
MPO-ANCA positive	45 (56.25%)	19 (38.78%)	26 (83.87%)	< 0.001*
MPO-ANCA titer, EU	141 (72, 200) (n = 45)	104 (53, 165) (n = 19)	170 (105, 200) (n = 26)	0.015*
PR3-ANCA positive	2 (2.50%)	2 (4.08%)	0 (0.00%)	0.519
PR3-ANCA titer, EU	69 (38, 69) (n = 2)	69 (38, 69) (n = 2)	0	-
RF, IU/mL	55.9 (22.7, 211.0)	26.7 (6.8, 442.9)	113.1 (53.5, 207.8)	0.157
ANA positive	45 (56.25%)	30 (61.22%)	15 (48.39%)	0.355
CCP positive	8 (10.00%)	8 (16.33%)	0 (0.00%)	0.020*
SSA positive	9 (11.25%)	6 (12.24%)	3 (9.68%)	1.000
SSB positive	4 (5.00%)	2 (4.08%)	2 (6.45%)	0.639
Jo-1 positive	1 (1.25%)	1 (2.04%)	0 (0.00%)	1.000
Scl-70 positive	2 (2.50%)	1 (2.04%)	1 (3.23%)	1.000
*Pulmonary function tests*				
FEV1, % predicted (n = 75)^**#**^	82.35 ± 17.32	84.03 ± 16.93	79.36 ± 17.92	0.265
FVC, % predicted (n = 75)^**#**^	81.85 ± 18.42	83.84 ± 19.10	78.30 ± 16.89	0.213
TLC, % predicted (n = 75)^**#**^	77.59 ± 13.43	79.53 ± 13.55	74.16 ± 12.73	0.096
DLCO, % predicted (n = 75)^**#**^	60.12 ± 15.53	62.12 ± 14.09	56.56 ± 17.52	0.138

Data are presented as median (IQR) or mean ± SD or No. (%)

^*^p < 0.05

^#^Seventy-five patients underwent pulmonary function tests at initial presentation, but the remaining 5 could not complete the tests because of the severity of their disease

ANCA: anti-neutrophil cytoplasmic antibody; IIP: idiopathic interstitial pneumonia; MPA: microscopic polyangiitis; ILD: interstitial lung disease; WBC: white blood cell; NEUT; neutrophil; LY: lymphocyte; HGB: haemoglobin; PLT: platelet; ESR: erythrocyte sedimentation rate; CRP: C reactive protein; PaO2: arterial oxygen tension; Cr: creatinine; MPO: myeloperoxidase; PR3: proteinase 3 antibody; RF: rheumatoid factor; ANA: antinuclear antibody; CCP: anti-cyclic citrullinated peptide antibody; SSA: anti-Ro; SSB: anti-La; Jo-1: anti-Jo-1; Scl-70: anti-Scl-70; FEV1: forced expiratory volume in 1 s; FVC: forced vital capacity; TLC: total lung capacity; DLCO: diffusing capacity of lung for carbon monoxide

Of the 80 patients with ANCA antibodies, 69 had a perinuclear ANCA pattern (pANCA), and 11 had a cytoplasmic ANCA pattern (cANCA). Forty-five patients had MPO antibodies, 2 had PR3 antibodies, and 1 was positive for both types. Seventeen of the 80 (21.25%) ANCA-positive patients had other autoimmune antibodies, including positive findings for ANA, SSA, Jo-1, Scl-70 and CCP, but did not meet the established criteria for a distinct CTD.

### Pulmonary function tests

Seventy-five of the 80 patients performed PFTs at initial presentation; the remaining 5 could not complete the tests because of the severity of the disease. There was no significant difference in the baseline PFTs between the groups (Table 2).

### Radiologic and histopathologic features

The major HRCT features of ANCA-positive ILDs patients included ground-glass opacities (86.25%), reticular patterns (61.25%), interlobular septal thickening (48.75%) and traction bronchiectasis (45.00%). Findings compatible with nonspecific interstitial pneumonia (NSIP) were the most common (63.75%). There were no significant differences in either abnormalities or HRCT patterns between MPA-ILDs group and ANCA-IIPs group (Table 3).

**Table 3:** Chest HRCT findings of patients with ANCA-IIPs versus patients with MPA-ILDs

HRCT findings n (%)	Combined (N = 80)	ANCA-IIPs (N = 49)	MPA-ILDs (N = 31)	*P value*
*Abnormalities*				
Ground-glass opacity	69 (86.25%)	44 (89.80%)	25 (80.65%)	0.322
Consolidation	8 (10.00%)	4 (8.16%)	4 (12.90%)	0.704
Traction bronchiectasis	36 (45.00%)	20 (40.82%)	16 (51.61%)	0.366
Honeycombing	19 (23.75%)	8 (16.33%)	11 (35.48%)	0.062
Reticular pattern	49 (61.25%)	29 (59.18%)	20 (64.52%)	0.648
Pulmonary artery dilation	4 (5.00%)	1 (2.04%)	3 (9.68%)	0.293
Curved linear opacity	17 (21.25%)	12 (24.49%)	5 (16.13%)	0.416
Pleural thickening	13 (16.25%)	9 (18.37%)	4 (12.90%)	0.556
Interlobular septal thickening	39 (48.75%)	23 (46.94%)	16 (51.61%)	0.819
Micronodular pattern	11 (13.75%)	6 (12.24%)	5 (16.13%)	0.742
Subpleural bulla	20 (25.00%)	10 (20.41%)	10 (32.26%)	0.292
Enlarged mediastinal lymph node	5 (6.25%)	3 (6.12%)	2 (6.45%)	1.000
*HRCT patterns*				
UIP	7 (8.75%)	3 (6.12%)	4 (12.90%)	0.421
NSIP	51 (63.75%)	33 (67.35%)	18 (58.06%)	0.477
Unclassifiable	22 (27.5%)	13 (26.53%)	9 (29.03%)	1.000

Data are presented as No. (%)

HRCT: high-resolution computed tomography; ANCA: anti-neutrophil cytoplasmic antibody; IIP: idiopathic interstitial pneumonia; MPA: microscopic polyangiitis; ILD: interstitial lung disease; UIP: usual interstitial pneumonia; NSIP: nonspecific interstitial pneumonia

In the MPA-ILDs group, 1 had surgical lung biopsy consistent with UIP pattern. 3 patients had renal biopsies conforming rapidly progressive glomerulonephritis. In the ANCA-IIPs group, 2 had surgical lung biopsies showing a NSIP pattern. All the 3 surgical lung biopsies were reviewed by 2 pathologists independently and found no evidence of capillaritis or vasculitis.

### Outcomes

After a median follow-up time of 40 (IQR 27–58) months, 15 of the 80 (18.75%) patients had died from all causes, and 1 (1.25%) patient had undergone lung transplantation. In the ANCA-IIPs group, 1 patient developed MPA based on pathological conformation (segmental necrotizing glomerulonephritis on kidney biopsy).

**Fig. 2:**
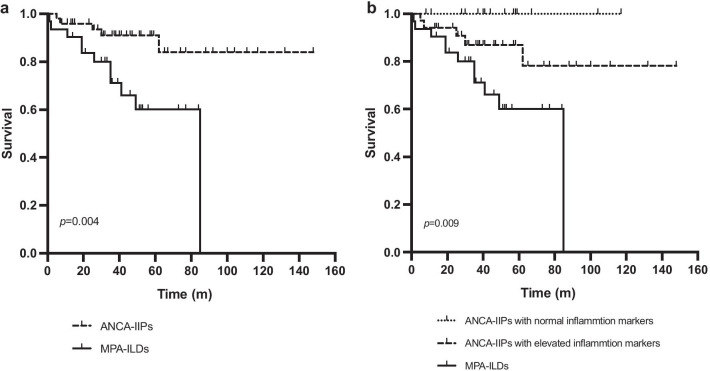
Kaplan–Meier survival curves. **a** Kaplan–Meier curves comparing survival time in patients with ANCA-IIPs versus patients with MPA-ILDs. The log-rank test showed a significant difference in survival (*p* = 0.004). **b** Kaplan–Meier curves comparing survival time in patients with ANCA-IIPs (stratified by inflammation marker levels) versus patients with MPA-ILDs. The log-rank test showed a significant difference in survival among these groups (*p* = 0.009). ANCA: anti-neutrophil cytoplasmic antibody; IIP: idiopathic interstitial pneumonia; MPA: microscopic polyangiitis; ILD: interstitial lung disease

In the univariable Cox hazards model, older age (hazard ratio [HR] 1.075, 95% confidence interval [95%CI] 1.011–1.043, *p* = 0.021), a diagnosis of MPA (HR 4.310, 95%CI 1.464–12.692, *p* = 0.008), higher ESR (HR 1.028, 95%CI 1.012–1.044, *p* = 0.001), honeycombing on HRCT (HR 3.264, 95%CI 1.203–8.858, *p* = 0.020) and %FVC predicted < 80% (HR 3.554, 95%CI 1.144–11.041, *p* = 0.028) were found to be negative prognostic factors among all ANCA-positive patients (Table 4). Kaplan–Meier survival curves demonstrated that the 5-year cumulative survival rate was 90.90% in the ANCA-IIPs group and 60.00% in the MPA-ILDs group (*p* = 0.004 by log-rank test) (Fig. 2a). Elevation of ESR was independently associated with a poor prognosis in multivariable analysis (HR 1.028, 95%CI 1.012–1.044, *p* = 0.001) (Table 4). To further analyse the effect that inflammation markers had on mortality, we divided the patients in the ANCA-IIPs group into patients with normal inflammation markers and elevated inflammation markers based on the value of ESR and CRP (ESR ≧20 mm/h and/or CRP ≧10 mg/L). The 5-year cumulative survival rates in these two subgroups of ANCA-IIPs patients and in the MPA-ILDs group were 100.00%, 86.90%, and 60.00%, respectively, which were significantly different (*p* = 0.009) (Fig. 2b).

**Table 4:** Cox hazard analysis of the risk of all-cause mortality in ANCA-positive ILDs patients

Variable	Univariable cox regression			Multivariable cox regression		
	HR	95% CI	*p* value	HR	95% CI	*p* value
Age, y	1.075	1.011–1.143	0.021*			
ESR, mm/h	1.028	1.012–1.044	0.001*	1.028	1.012–1.044	0.001*
Honeycombing	3.264	1.203–8.858	0.020*			
MPA	4.310	1.464–12.692	0.008*			
%FVC predicted < 80%^**#**^	3.554	1.144–11.041	0.028*			

*p < 0.05

^**#**^For the 5 patients who did not complete the pulmonary function tests, their data were interpolated using the mean substitution method according to their group

ANCA: anti-neutrophil cytoplasmic antibody; ILD: interstitial lung disease; ESR: erythrocyte sedimentation rate; MPA: microscopic polyangiitis; FVC: forced vital capacity

## Discussion

This study retrospectively analysed the clinical, laboratory, radiologic and prognostic features of a group of 80 patients with ILDs and positive serum ANCA from a single centre, with a focus on the differences between the MPA-ILDs group and the ANCA-IIPs group. To our knowledge, this study collected the largest number of patients with ANCA-positive ILDs to date. Compared with patients with ANCA-IIPs, patients in the MPA-ILDs group had a greater degree of systemic inflammation, including a higher incidence of fever and elevated inflammation markers. The survival of the MPA-ILDs patients was lower than that of the ANCA-IIPs group, and further stratified analysis demonstrated that patients with elevated inflammation markers in the ANCA-IIPs group had a worse prognosis than those with normal inflammation markers.

Studies concerning the relationship of ANCA, AAV and ILDs are still limited. The current study showed that a small proportion of patients with ILDs were ANCA-positive, and some of them were related to AAV. Prior studies found that ANCA positivity is seen in approximately 4.02–8.80% of patients with IPF [13, 14, 17, 18] and 4.44–7.73% of patients with IIPs [17, 19, 20] at the time of initial diagnosis. Similarly, our results showed that 4.60% of all the ILDs patients and 4.50% of IIPs patients were ANCA-positive at first diagnosis. The current diagnostic algorithm for ILDs suggests screening autoantibodies related to rheumatoid arthritis, Sjögren syndrome, dermatomyositis and polymyositis and systemic sclerosis for any underlying causes but does not emphasize screening ANCA or AAV [2, 5, 11, 12]. Therefore, MPA-ILDs patients with mild or occult onset extrapulmonary involvement are easily classified as IIPs or IPF by mistake. In addition, patients with isolated ANCA-positive ILDs are now classified as IIPs or IPF, although they share similar features with IPAF, i.e., positive antibodies but lacking extrapulmonary manifestations. These patients should be distinguished from those with IIPs. Therefore, screenings for ANCA and evaluations of underlying vasculitis should be considered in all patients presenting with ILDs, as suggested by recent IPF guidelines [21].

Consistent with the literature concerning IPF patients [14], we found that 2.63% of patients with ANCA-negative IIPs seroconverted to positive during follow-up. In our study, only 1/34 (2.94%) patient in the initial ANCA-IIPs group developed MPA during follow-up. This was relatively lower than previous studies, in which AAV development was seen in 27.78% of MPO-ANCA–positive IPF patients [13] and 34.62% of MPO-ANCA–positive IIPs patients [19]. Possible reasons might be the use of steroids and immunosuppressants preventing the development of systemic vasculitis and the limited duration of follow-up. Of note, the potential to develop MPA was observed not only in ANCA-positive IIPs patients but also in 3/723 (0.41%) ANCA-negative IIPs patients, indicating that ILDs can be the initial presentation of MPA, similar to CTD [10, 22]. Therefore, close follow-up is needed to monitor systemic vasculitis development in patients with an initial IIPs diagnosis, regardless of their ANCA testing results. On the other hand, ILDs patients with isolated ANCA positivity might represent a limited pulmonary form of AAV, a type of organ-limited vasculitis [23]. However, previous lung biopsies showed no histopathologic evidence of capillaritis or vasculitis in ANCA-positive ILDs patients [13, 24, 25]. More evidence is needed to validate this concept.

Accumulating evidence suggests an association among ANCA, MPA and IPF [13, 14, 24]. A total of 4.02–12.90% of the patients initially diagnosed with IPF were ANCA-positive either upon first diagnosis or during follow-up, and 13.85–25.00% of these patients developed AAV [13, 14, 24]. Furthermore, usual interstitial pneumonia (UIP) appeared to be the predominant HRCT pattern in AAV-ILD and MPA-ILD [10, 26]. However, in this study, the UIP pattern was seen in only 6.12% and 12.90% of patients in the ANCA-IIPs group and MPA-ILDs group, respectively. Only a few studies have explored the association among ANCA, MPA and IIPs. One study found that 6.40% of the patients with non-IPF (NSIP, COP and unclassifiable IIPs) were MPO-ANCA–positive, and 27.27% of them developed MPA [19]. Our findings and previous studies revealed that ANCA-positive ILDs is a heterogeneous group of patients consisting of not only UIP but also many other ILDs patterns. Further studies are warranted to elucidate the relationship between ANCA and non-IPF ILDs.

There is a lack of large-sample comparisons regarding the clinical differences between ANCA-IIPs and MPA-ILDs. A previous study found that among patients with ANCA-positive IIPs, compared with patients who did not develop MPA, patients who developed MPA had a significantly higher frequency of UIP patterns on HRCT, but there were no significant differences in clinical features, laboratory tests, PFTs and 5-year cumulative survival rates [19]. The current study showed that patients in the MPA-ILDs group were older and had a more severe systemic inflammatory response, a higher proportion of fever and higher levels of inflammatory markers than patients in the ANCA-IIPs group. Although honeycombing on HRCT tended to appear in a larger proportion in the MPA-ILDs group, the difference did not reach statistical significance.

The prognosis of ANCA-positive ILDs are not clear. There have been no consistent findings about the effect of ANCA positivity on prognosis in ILDs patients. Some studies showed a higher mortality rate in patients with ANCA-positive IPF than in patients with ANCA-negative IPF [13, 15], while some studies found no difference [14]. Our study revealed that two classes of factors determined the outcome of ANCA-positive ILDs patients. First, features related to ILDs, including a lower FVC level at initial diagnosis and honeycombing on HRCT, were associated with higher mortality. This finding is consistent with prior studies [14, 19]. Second, systemic involvement, including a diagnosis of vasculitis and higher systemic inflammation, may indicate poor outcomes. Multivariable analysis further confirmed that the ESR level was an independent risk factor for mortality. Therefore, we infer that patients with ANCA-positive ILDs might represent a different entity from ILDs, since systemic inflammatory response were the determinant factor for mortality.

In addition, there has been no direct comparison in survival between MPA-ILDs and isolated ANCA-positive ILDs. Our results showed that MPA-ILDs group had significantly higher mortality than ANCA-IIPs group and further stratified analysis revealed that ANCA-IIPs patients with elevated ESR/CRP had a worse prognosis than those with normal inflammation markers. Hence, stratified treatment should be considered in the management of patients with ANCA-positive ILDs. Patients with MPA-ILDs, who have the worst prognosis, should be treated aggressively with systemic steroids in combination with other immunosuppressants, as suggested for MPA [27]. Isolated ANCA-positive IIPs with elevated levels of ESR/CRP are associated with a relatively poor prognosis and should be distinguished from IIPs. Systemic steroids with or without other immunosuppressants may be appropriate under these circumstances. For isolated ANCA-positive IIPs with normal inflammation markers, initial treatment could be conservative, similar to IIPs or IPF, but close follow-up is essential to monitor signs of development of systemic vasculitis for any adjustment in treatment.

This study had several limitations. First, it was a retrospective study. Selection bias is possible because we only included patients with available serologic results. Second, because of the fact that it was conducted in a single tertiary referral centre and the small sample size of ANCA-positive patients, the results of the multivariable analysis of risk factors for prognosis should be carefully interpreted. More studies are needed to confirm our findings.

## Conclusions

In conclusion, patients with MPA-ILDs had a greater degree of systemic inflammation than patients with isolated ANCA-positive IIPs. Among patients with ANCA-positive ILDs, the prognoses of ANCA-IIPs with normal inflammation markers, ANCA-IIPs with elevated inflammation markers and MPA-ILDs were sequentially worse. Therefore, stratified treatment should be considered in the management of ILDs patients positive for ANCAs.

## Supplementary Information

**Additional file1: Figure S1.** High-resolution computed tomography images of the two major patterns of interstitial lung disease associated with ANCA. Representative features of usual interstitial pneumonia (UIP) pattern are shown in a: honeycombing and traction bronchiectasis with basal and subpleural predominance. UIP pattern was confirmed by surgical lung biopsy. Nonspecific interstitial pneumonia (NSIP) pattern, characterized by diffused ground-glass opacities and reticular opacities, is depicted in b, which was confirmed by lung histopathology.

## Data Availability

This study was registered in ClinicalTrials.gov (NCT04413149). The datasets generated and analysed during the current study are available in the ClinicalTrials repository [https://clinicaltrials.gov/ct2/show/NCT04413149?term=NCT04413149&draw=2&rank=1].

## Abbreviations

AAV: Anti-neutrophil cytoplasmic antibody-associated vasculitis
ANA: Antinuclear antibody
ANCA: Anti-neutrophil cytoplasmic antibody
CCP: Cyclic citrullinated peptide antibody
CRP: C-reactive protein
CTD: Connective tissue disease
DLCO: Diffusing capacity of lung for carbon monoxide
ESR: Erythrocyte sedimentation rate
FEV1: Forced expiratory volume in 1s
FVC: Forced vital capacity
HRCT: High-resolution computed tomography
IIP: Idiopathic interstitial pneumonia
ILD: Interstitial lung disease
IPAF: Interstitial pneumonia with autoimmune features
IPF: Idiopathic pulmonary fibrosis
MPA: Microscopic polyangiitis
MPO: Myeloperoxidase
NSIP: Nonspecific interstitial pneumonia
PaO2: Arterial oxygen tension
PFT: Pulmonary function test
PR3: Proteinase 3
TLC: Total lung capacity
UIP: Usual interstitial pneumonia
