# Supplementary material for: Clinical features and long-term outcomes of interstitial lung disease with anti-neutrophil cytoplasmic antibody
Source: BMC Pulm Med. 2021 Mar 16;21:88. doi: 10.1186/s12890-021-01451-4 (PMC7968287; doi:10.1186/s12890-021-01451-4)
Supplement: Supplementary file 1 — Additional file1: Figure S1. High-resolution computed tomography images of the two major patterns of interstitial lung disease associated with ANCA. Representative features of usual interstitial pneumonia (UIP) pattern are shown in a: honeycombing and traction bronchiectasis with basal and subpleural predominance. UIP pattern was confirmed by surgical lung biopsy. Nonspecific interstitial pneumonia (NSIP) pattern, characterized by diffused ground-glass opacities and reticular opacities, is depicted in b, which was confirmed by lung histopathology. [file 12890_2021_1451_MOESM1_ESM.pdf]

Clinical features and long-term outcomes of interstitial lung disease with anti-neutrophil cytoplasmic antibody

Xin Sun[1*], Min Peng[1*], Ting Zhang[1], Zongru Li[2], Lan Song[3], Mengtao Li[4], Juhong Shi[1]

*These authors contributed equally to this work.

CORRESPONDENCE AUTHOR

Juhong Shi, M.D., Department of Respiratory and Critical Care Medicine, Peking Union Medical College Hospital, Chinese Academy of Medical Science & Peking Union Medical College, No. 1 Shuai Fu Yuan, Dongcheng District, Beijing 100730, China

Tel: 86-13701178492

Fax: 86-10-69155028

E-mail: shijh@pumch.cn

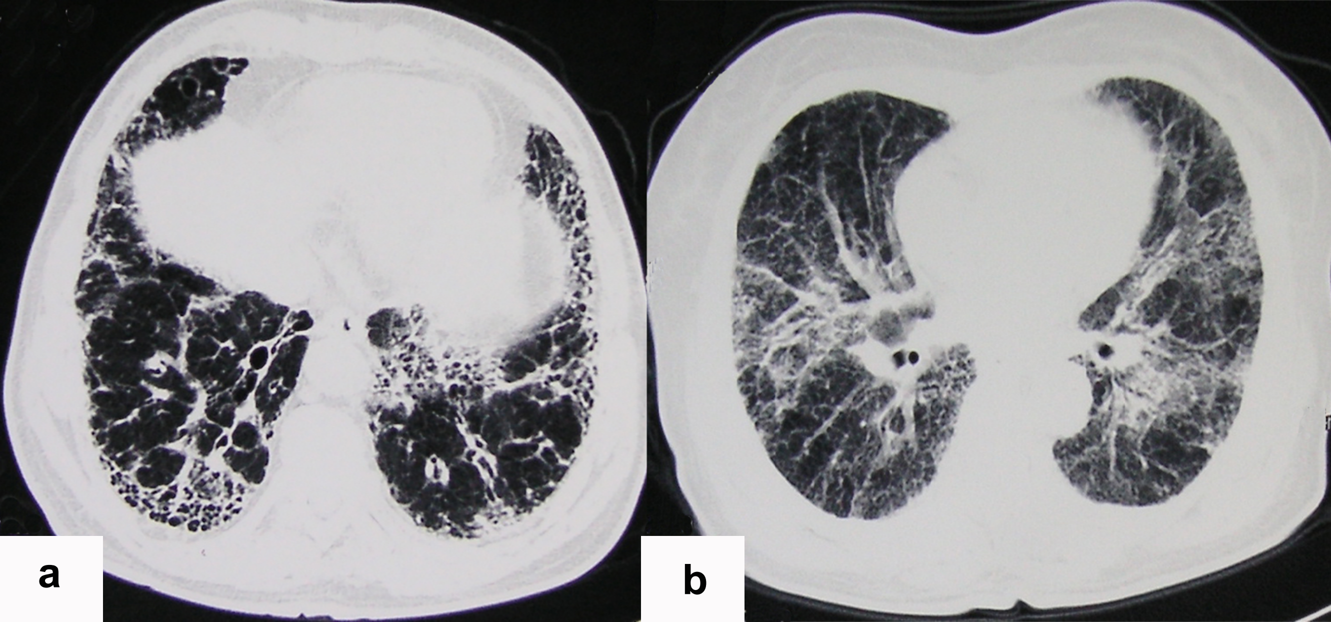
a
b
